# Parameter sensitivity analysis and structural optimization of PLC construction method pile cofferdam based on RSM

**DOI:** 10.1371/journal.pone.0339267

**Published:** 2025-12-23

**Authors:** Zhongchu Tian, Ye Dai, Shiyao Wang, Wei Zhang

**Affiliations:** 1 School of Civil and Environmental Engineering, Changsha University of Science & Technology, Changsha, Hunan, China; 2 School of Civil Engineering, Fujian University of Technology, Fuzhou, Fujian, China; 3 Nanning Branch of China ANENG Group First Engineering Co., Ltd., Nanning, Guangxi, China; Institute of Infrastructure Technology Research and Management, INDIA

## Abstract

To explore the influence patterns of various structural parameters of the PLC construction method pile cofferdam on its mechanical properties, and to optimize the structural design for enhancing engineering safety and economy, this study relied on the PLC construction method pile cofferdam of the Luoxizhou Super Bridge. Multiple sensitivity analysis methods were employed to identify sensitive parameters. Subsequently, a multi-objective optimization study was conducted based on the identified sensitive parameters. Additionally, the entropy weight TOPSIS decision-making approach was introduced to quantify the advantages and disadvantages of each optimized scheme and the initial design scheme. The results indicate that: (1) Sensitivity rankings derived from the single parameter method and the response surface methodology are consistent across parameters, but the response surface methodology provides more accurate sensitivity quantification. (2) The thicknesses of the steel sheet pile wall, steel pipe pile wall, and bottom-sealing concrete significantly affect the maximum displacement and maximum stress of the cofferdam. (3) The entropy-weighted TOPSIS decision-making can effectively evaluate the advantages and disadvantages of the Pareto solution set optimized by the NSGA-II algorithm. (4) Economic cost and structural performance exhibit a nonlinear synergy: As cost increases, the maximum displacement of the cofferdam decreases markedly, whereas the maximum stress of the bottom-sealing concrete decreases at a slower rate.

## 1. Introduction

As bridge construction progresses into deeper waters with larger spans, traditional steel sheet pile cofferdams and steel pipe pile cofferdams have increasingly encountered stability issues and inadequate deformation control under complex geological conditions, including strong water flow and weak soil layers [1–3] These limitations compromise their suitability for modern bridge construction requirements. To address these challenges, a PLC (Pipe-Sheet Pile Composite) construction method pile cofferdam has been developed, combining the advantages of both traditional structures. The PLC construction method pile cofferdam establishes a spatial, cooperative force-transfer system by creating a rigid connection between the special locking buckle on the steel pipe pile and the Larsen steel sheet pile. This structure efficiently withstands the combined effects of hydrodynamic pressure and water-soil pressure encountered in deep-water environments. Following dewatering and the placement of bottom-sealing concrete, it provides a highly stable working platform for constructing bridge substructures, significantly improving adaptability under complex working conditions. Compared with traditional steel cofferdams, the PLC construction method pile cofferdam also effectively reduces overall steel consumption, achieving significant cost-effectiveness while ensuring structural safety. However, as a novel composite structure, its mechanical performance is influenced by multiple factors. To accurately evaluate its behavior in complex geological conditions, a sensitivity analysis of key parameters is essential. This analysis will explore the influence of various factors on its mechanical properties, thereby providing a robust theoretical basis and technical support for optimizing design, improving construction quality, and ensuring bridge construction safety.

Parameter sensitivity analysis has evolved from single parameter method to response surface methodology. The latter effectively quantifies the interaction effects of multiple parameters on complex structures by constructing explicit response equations. For instance, Liu J et al. [4] established importance classification for cable-stayed bridge design parameters using the response surface methodology. Zhan Y et al. [5] investigated the influence of design parameters on the structural response of an asymmetric steel truss cable-stayed bridge. Hooshmandi S et al. [6] employed the response surface methodology combined with nonlinear finite element analysis results to examine the effects of design variables (such as reinforcing bar diameter, concrete compressive strength, and reinforcing bar yield strength) on the seismic performance of RC pier walls. Chen F et al. [7] conducted a comprehensive investigation into the causes of cracking and deflection in PC continuous box girder bridges, as well as the weight of the influencing factors. Against the backdrop of a long-span, single-tower hybrid girder cable-stayed bridge, Hu H et al. [8] explored the impact of deck crane parameters on the mechanical properties of the main girder.

However, sensitivity studies of cofferdam structures still predominantly employ the single parameter method. For instance, Mao J et al. [9] examined the impact of the thickness of the steel box cofferdam wall, the height of the water injection between the double walls, and the spacing of the support rods within the double walls on the structure’s mechanical state via numerical simulation. Based on simulation results from a Midas finite element model, Du G et al. [10] explored the influence of design parameters on cofferdam deformation, providing an accurate analysis of the deformation and stress variation in the cofferdam and surrounding soil under various construction conditions. Wang Q et al. [11] systematically studied the quantitative relationship between the distance separating the foundation pit and the cofferdam and its influence on foundation pit excavation. Their work revealed the deformation patterns and stability mechanisms of the cofferdam under different design schemes. Li P et al. [12] systematically investigated the influence of tidal levels, soil parameters, support stiffness, and other parameters on the lateral deflection of super-long steel sheet pile cofferdams. They also elucidated the interrelationships between these various factors and cofferdam deformation.

Meanwhile, scholars optimize structures based on determined sensitivity parameters. For example, Jiang Z et al. [13] proposed a multi-objective optimization model using an improved Sparrow Search Algorithm, addressing the lack of systematic evaluation methods for steel pipe pile cofferdam construction schemes. Their model aimed to minimize both construction duration and cost while enhancing structural performance. Shao J et al. [14] developed a multi-objective optimization method combining the response surface methodology with particle swarm optimization, examining variations in optimal design parameters and structural responses relative to cost in double-wall steel cofferdams for deep-water bridge foundations. Wan Y [15] optimized the internal support system of the PLC construction method pile cofferdam, significantly enhancing its structural safety and cost-effectiveness. However, Most existing research focuses on traditional cofferdam structures, with few studies examining the sensitivity analysis and multi-objective optimization of PLC construction method pile cofferdam based on the response surface methodology.

Based on the PLC construction method pile cofferdam used in the Luoxizhou Super Bridge project, this study employed the response surface methodology (RSM) to analyze structural sensitivity and investigate the influence of design parameters on structural stress distribution and deformation characteristics. A single parameter analysis approach was implemented to validate the sensitivity results obtained through Response surface methodology, with the objective of cross-verifying the reliability of the response surface-derived sensitivity assessments. Subsequent to parameter sensitivity identification, a collaborative optimization model combining response surface methodology and NSGA-II was developed to execute multi-objective optimization. This model aims to simultaneously enhance structural safety and economic efficiency in practical engineering applications. Furthermore, the Entropy-weighted TOPSIS method was employed to quantitatively evaluate the Pareto optimal solutions and design value, providing theoretical support for similar engineering practices.

## 2. Fundamental theory

### 2.1. Response surface methodology

Response Surface Methodology employs regression analysis of finite experimental datasets to fit response functions $\overset{―}{y}(x)$ , converting the implicit relationship between a structure’s actual response y(x) and its design parameters $x=(x_{1},x_{2},\cdots ,x_{n})$ into an explicit approximation [16–19]. Given the demonstrated capability of quadratic polynomials to accurately characterize nonlinear effects, this study utilized Central Composite Design (CCD) to establish a quadratic response surface model. This approach significantly reduces the number of required tests while ensuring convergence and enables efficient analysis of multifactor interactions through coordinated configurations of axial and center points. The functional expression is as follows:


$$\overset{―}{y}(x)=a+\sum_{i=1}^{n}b_{i}x_{i}+\sum_{i=1}^{n}\sum_{j=1}^{n}c_{ij}x_{i}x_{j}$$ (1)

In the established formula: $\overset{―}{y}(x)$ represents the response function; a、 $b_{i}$ and $c_{ij}$ denote undetermined coefficients; while $x_{i,j}(i,j=1,2,3,\cdot \cdot \cdot ,n)$ signifies the parameters requiring identification.

At the same time, Criteria $R^{2}$ and $R_{adj}^{2}$ are introduced to verify the model’s accuracy. Criterion $R^{2}$ represents the model’s explanatory power over the data. The closer its value is to 1, the higher the model’s accuracy. However, this value may increase as the number of independent variables grows. However, this metric may demonstrate a false increase as the number of independent variables increases. Criterion $R_{adj}^{2}$ accounts for the influence of parameter quantity, thereby avoiding overfitting. If both values are close to 1 and their difference is minimal, the model can be considered highly accurate.

### 2.2. Parameter sensitivity analysis

The objective of parameter sensitivity analysis is to quantify the sensitivity of each parameter to the structural response. Common methods include the single parameter method and the response surface methodology.

#### 2.2.1. Sensitivity analysis based on single parameter methodology.

In sensitivity analysis using the single parameter method, the value of one variable is altered at a time while other parameters are held constant to observe variation of change in structural responses. The sensitivity coefficient is used to characterize it quantitatively, and the calculation formula is as follows:


$$I_{i}=\frac{\Delta y_{i}/y_{i}}{\Delta x_{i}/x_{i}}$$ (2)

In the formula: $I_{i}$ denotes the parameter sensitivity coefficient; $y_{i}$ and $\Delta y_{i}$ represent the baseline value and incremental value of structural response, respectively; $x_{i}$ and $\Delta x_{i}$ represent the baseline value and incremental value of the parameter, respectively.

#### 2.2.2. Sensitivity analysis based on response surface methodology.

Sensitivity analysis based on the response surface methodology involves calculating partial derivatives of the response surface surrogate model to derive sensitivity coefficients for each parameter, thereby enabling quantitative assessment of how uncertainty factors influence structural responses. The computational formulation is presented as follows:


$$S(x_{i})=\left(\frac{\Delta \overset{―}{y}(x_{i})}{\overset{―}{y}(x_{i})}\right)/\left(\frac{\Delta x_{i}}{x_{i}}\right)=|\overset{―}{y^{\prime }}(x_{i})|\frac{x_{i}}{\overset{―}{y}(x_{i})}$$ (3)

In the formula: $S(x_{i})$ is the parameter sensitivity factor; $\overset{―}{y}(x_{i})$ and $\Delta \overset{―}{y}(x_{i})$ denote the value and increment of the response function $\overset{―}{y}(x)$ at point $x_{i}$, respectively.

Concurrently, the sensitivity percentage is introduced to quantify the sensitivity level of each parameter more intuitively. The calculation formula is defined as follows:


$$\gamma_{x_{k}}=\frac{\left|S(x_{k})\right|}{\sum_{i}^{n}\left|S(x_{i})\right|}\times 100\%$$ (4)

In the formula: $\gamma_{x_{k}}(k\in i)$ represents the sensitivity percentage of parameter $x_{k}$ in the sensitivity analysis of the structural response.

The sensitivity factor $S(x_{i})$ and sensitivity percentage $\gamma_{x_{i}}$ are dimensionless real numbers. Larger absolute values indicate greater sensitivity of the objective function $\overset{―}{y}(x)$ to the parameter $x_{i}$. Parameter sensitivity is categorized into four classes based on sensitivity percentage, with specific criteria shown in Table 1.

**Table 1:** Classification of parameter importance levels.

The influence degree of parameters	Sensitivity percentage
Important parameter	[100%,50%]
Major parameter	(50%,25%]
Minor parameter	(25%,5%]
Negligible parameter	(5%,0%]

### 2.3. Multi-objective optimization based on the NSGA-II algorithm

Multi-objective optimization problems are prevalent in the field of bridge engineering. The essence of multi-objective optimization is to simultaneously optimize two or more conflicting objective functions. This involves finding the minimal or maximal values of a group of objective functions under given constraint conditions [20–22]. The mathematical formulation is as follows:


$$\begin{array}{c} {}*20c\min/\max f(x)=(f_{1}(x),f_{2}(x),\cdots ,f_{m}(x))\\ s.t.g_{j}(X)\leq 0,j=1,2,\cdots ,J\\ h_{k}(X)=0,k=1,2,\cdots ,K \end{array}\}$$ (5)

In the formulation: $x=(x_{1},x_{2},\cdots ,x_{n})$ is an *n*-dimensional decision variable; $f_{i}(x)$ represents the *i*-th objective function; $g_{j}(x)$ and $h_{k}(x)$ denote the inequality constraint and equality constraint, respectively; m is the number of objective functions; j and k are the numbers of inequality constraints and equality constraints, respectively.

Unlike single-objective optimization problems, multi-objective optimization problems typically do not possess a single global optimum. Instead, they yield a set of solutions known as Pareto optimal solutions. Pareto optimal solutions are those for which no other solutions in the solution space exist that can dominate them. The solution set forms the Pareto frontier, which characterizes the diversity of solutions and the trade-offs between objectives in multi-objective optimization.

**Fig 1:**
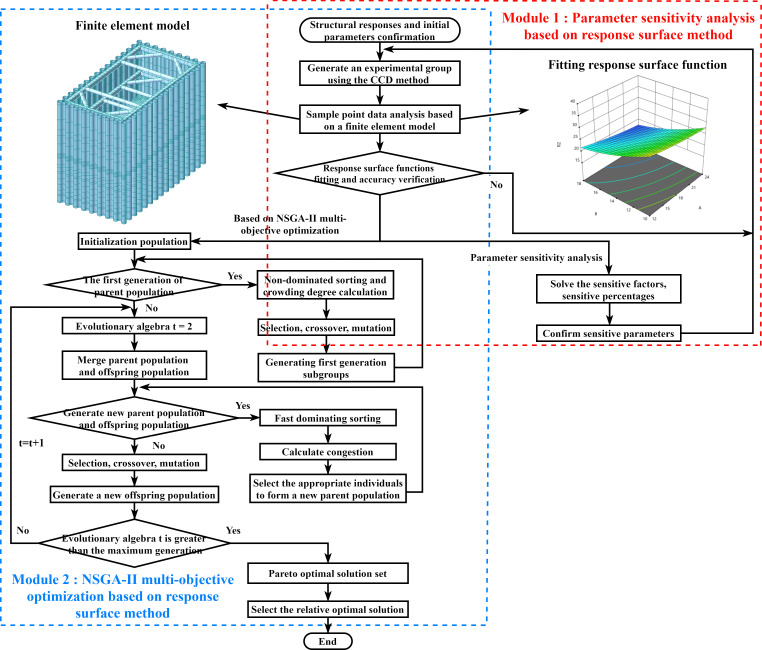
Flow chart of parameter sensitivity analysis and NSGA-II multi-objective optimization based on response surface methodology.

Given the complexity and multi-objective nature of the parameter optimization problem for the PLC construction method pile cofferdam, this paper employs the NSGA-II algorithm to find a solution set. This algorithm combines fast non-dominated sorting with an elite strategy to enhance the efficiency and solution quality. It enables direct acquisition of a Pareto optimal solution set, providing a comprehensive reference for decision-making. The NSGA-II algorithm is particularly well-suited for multi-objective optimization problems with conflicting objectives. The flow chart detailing the multi-objective optimization process based on NSGA-II is presented in Module 2 of Fig 1.

## 3. Engineering overview

### 3.1. Introduction of the project

**Fig 2:**
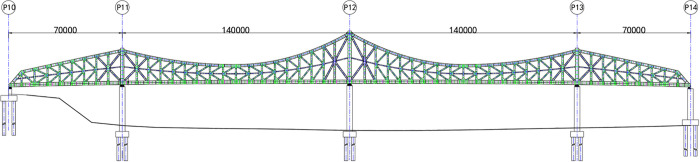
Elevation view showing the main bridge type arrangement of the Luoxizhou Super Bridge (unit: mm).

**Fig 3:**
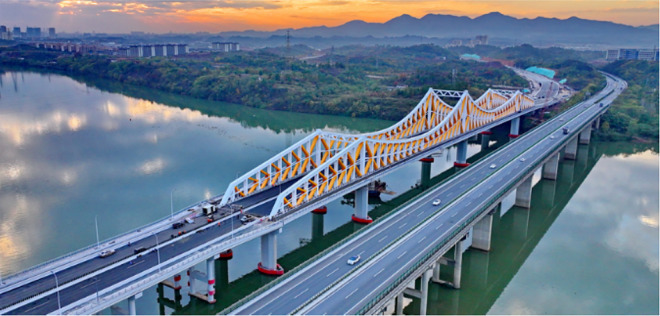
Luoxizhou Super Bridge in completed state.

The Luoxizhou Super Bridge is China’s first K-shaped steel truss bridge constructed via the incremental launching method. The span layout is 70 m + 140 m + 140 m + 70 m, resulting in a total length of 420 m and a standard width of 40.5 m. Fig 2 presents the elevation drawing of the main bridge type arrangement, and Fig 3 shows the completed bridge state.

**Fig 4:**
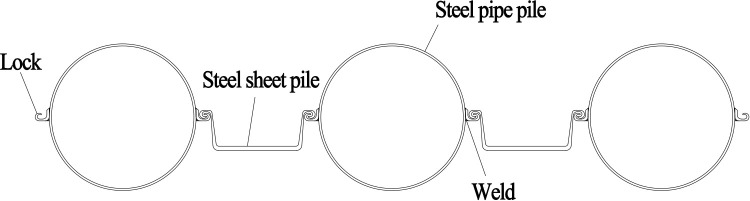
Structure schematic diagram of the PLC construction method pile cofferdam.

The substructure of the main bridge features double-column piers. The pier shafts have a collision-resistant spindle-shaped cross-section. The foundation system consists of pile caps supported by bored cast-in-place piles with a diameter of 2.2 meters. Among these, the in-water piers P11# to P14# were constructed using PLC construction method pile cofferdams. A structural schematic diagram of the PLC construction method pile cofferdam is provided in Fig 4.

**Fig 5:**
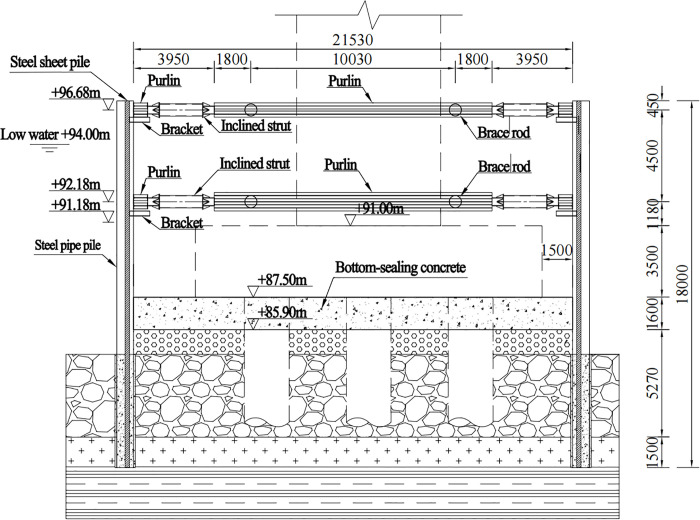
Structure elevation drawing of the PLC construction method pile cofferdam (unit: mm).

**Fig 6:**
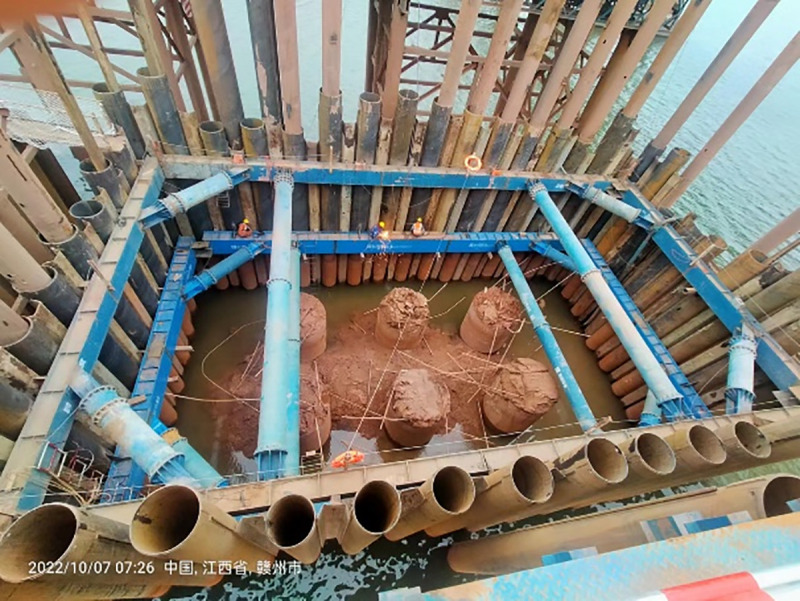
Site construction drawing of the PLC construction method pile cofferdam.

The structural system of the PLC construction method pile cofferdam in this project consists of a arrangement comprising Q345-grade steel pipe piles (Φ820mm×14 mm) and Q345-grade Lassen IVw steel sheet piles. The internal bracing framework employs Q235-grade steel pipes (Φ609mm×16 mm), while the purlin utilizes double-layer Q235B-grade 2 × H700 × 300 × 13 × 24 structural steel sections. The bottom-sealing concrete layer, with a thickness of 1.6 m, is constructed using C30-grade concrete. A minimum clear distance of 1.3 m is maintained between the pile cap and the cofferdam structure. During the construction phase, the normal water level is recorded at +94.11 m, with both flood regulation and design water levels reaching +96 m. The peak flow velocity during the flood season is 2.34 m/s. The structural elevation drawing and site construction drawing of the PLC construction method pile cofferdam are presented in Fig 5 and Fig 6, respectively.

### 3.2. Finite element model of the PLC construction method pile cofferdam

**Fig 7:**
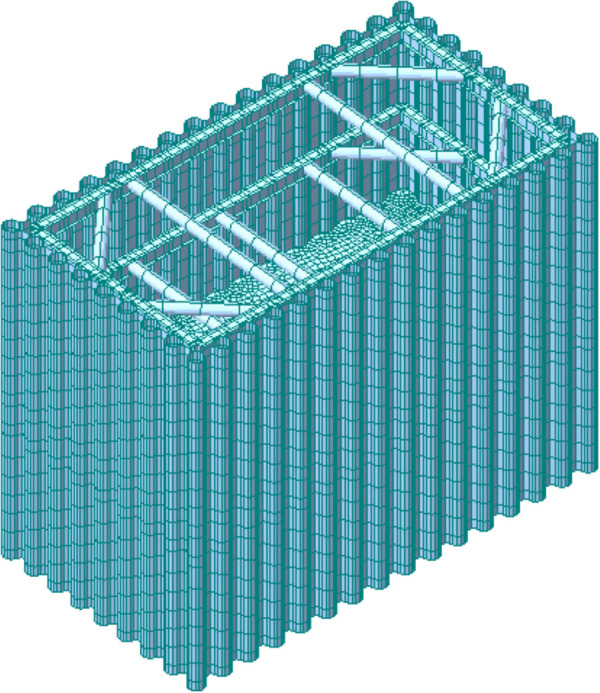
Finite element model of PLC construction method pile cofferdam.

A finite element model of the PLC construction method pile cofferdam at the Luoxizhou Super Bridge was created using Midas Civil software. The internal supports, the cofferdam wall panels, and the bottom-sealing concrete were modeled using beam elements, shell elements, and solid elements, respectively. The model materials were selected according to the actual design, with Q235 steel used for the internal bracings, Q345 steel for the cofferdam wall panels, and C30 concrete for the bottom-sealing concrete. The model comprised a total of 32,301 elements and 34,051 nodes. Elastic connections simulated the supporting action provided by the cushion blocks and corbels between the internal supports and the cofferdam wall panels. The bottom of the bottom-sealing concrete was fully fixed, while the bottom of the purlin wall panel was constrained only in the Z-direction. The m-method was employed to calculate the soil springs, which were then simulated through nodal elastic supports. Relevant loads were computed in accordance with specifications such as the General Code for Design of Highway Bridges and Culverts [23]. Based on the principle of separate calculation for water and soil pressures, the water pressure and soil pressure were calculated respectively. Subsequently, these pressures were applied as surface loads within fluid loads to the cofferdam wall panel, so as to accurately simulate the actual stress conditions experienced by the cofferdam. The finite element model is presented in Fig 7.

### 3.3. Evaluation of the accuracy of numerical predictions of structural response

During the construction of cofferdam foundations, the stress state of the supporting structure exhibits dynamic variations influenced by multiple factors, including flow velocity and hydrodynamic pressure. Meanwhile, the actual groundwater level is affected by numerous complex variables, making it challenging to accurately simulate its variation patterns using numerical models. The simplified treatment of complex geological conditions and multi-factor coupling during the modeling process also induced discrepancies between numerical predictions of structural responses and measured field data. To verify the model’s reliability and accuracy, numerical prediction results are systematically compared with field monitoring data.

According to the construction technology, the cofferdam calculated conditions for this project were categorized into the following three stages:

Stage 1: Following dewatering to the low water level (elevation +94 m), the steel casings interfering with internal support installation were dismantled. Temporary corbels were welded, and the first layer of purlins and internal supports were installed.

Stage 2: After cleaning the foundation pit bottom surface, underwater placement of bottom-sealing concrete was carried out.

Stage 3: After the bottom-sealing concrete attained the design strength, water was pumped to elevation +91.18 m, the second layer of purlins was installed, while removing the steel casings. At this stage, the steel cofferdam reached its maximum stress condition and was designated as the most unfavorable condition.

#### 3.3.1. Layout of monitoring points.

Based on Technical Standard for Monitoring of Building Foundation Pit Engineering, relevant literature, and construction experience [24], deformation monitoring and strain monitoring shall be conducted for the cofferdam supporting structure and bracing structure respectively.

**Fig 8:**
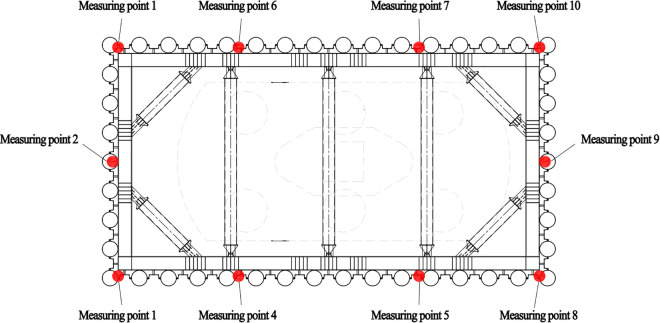
Layout diagram of deformation monitoring points for bracing structure of the PLC construction method pile cofferdam.

Deformation monitoring points were installed on the outer side of the cofferdam top. Specifically, 4 and 3 monitoring points were evenly distributed along the long and short sides of the cofferdam, respectively. At the intersections of the long and short sides, a shared monitoring point was established. Consequently, each cofferdam was equipped with a total of 10 deformation monitoring points. The layout diagram of deformation monitoring points for the PLC construction method pile cofferdam supporting structure at piers P11#-P14# is presented in Fig 8.

**Fig 9:**
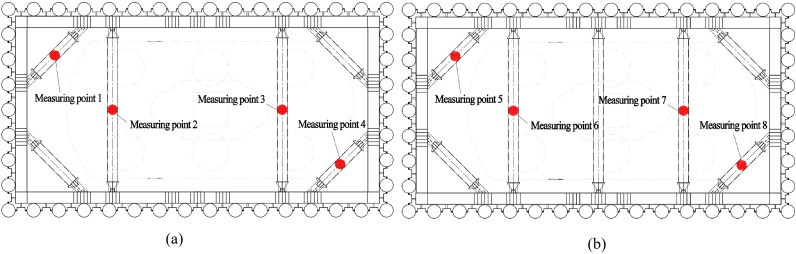
Layout diagram of stress monitoring points for supporting structure of the PLC construction method pile cofferdam. (a) The first layer. (b) The second layer.

To ensure the continuity and comparability of stress variations along the same vertical profile, the horizontal positions of measuring points on the upper and lower support structures must remain consistent when configuring strain monitoring points on the cofferdam supporting structure. The specific arrangement of strain monitoring measuring points follows the configuration illustrated in Fig 9.

#### 3.3.2. Data comparison.

**Fig 10:**
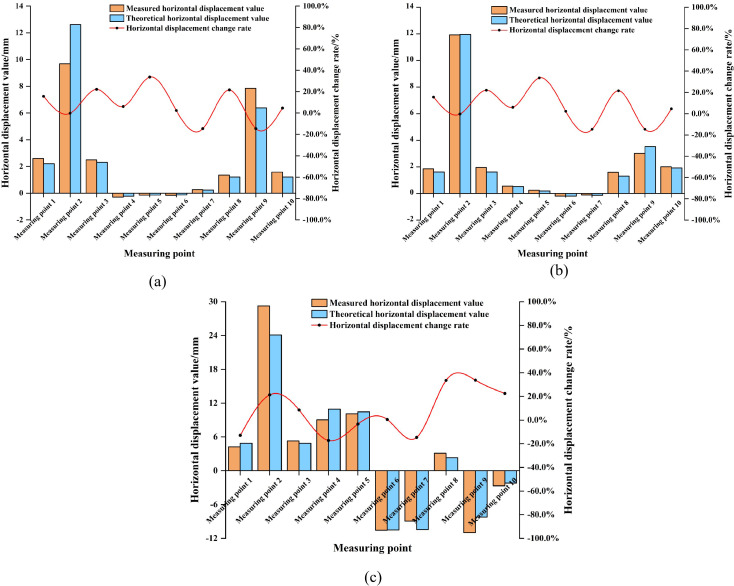
Comparison chart of numerical predictions and monitoring results for deformation of the bracing structure of the P11# pier PLC construction method pile cofferdam under different stages. (a) Stage 1. (b) Stage 2. (c) Stage 3.

**Fig 11:**
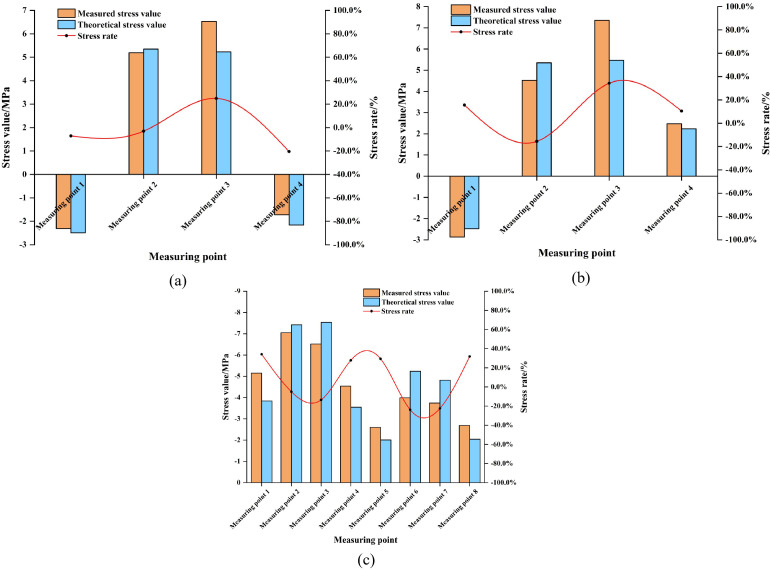
Comparison chart of numerical predictions and monitoring results for stress of the supporting structure of the P11# pier PLC construction method pile cofferdam under different stages. (a) Stage 1. (b) Stage 2. (c) Stage 3. Note: Due to construction stage limitations, only the first layer of the support structure was accessible for monitoring during the initial two stages. Four stress monitoring points were installed on this support structure. As construction progressed to working stage 3, the expansion of the work area enabled monitoring of the second layer of the support structure. An additional four stress monitoring points were installed on this second layer, which increased the total number of stress monitoring points to eight at this stage.

Due to space constraints, only the comparative plots of numerical predictions versus monitoring data for bracing structure deformations and supporting structure stresses in the PLC construction method pile cofferdam of P11# pier are presented. Detailed comparisons are shown in Figs 10 and 11.

As illustrated in Figs 10 and 11, the numerical simulation results of deformation for the PLC construction method pile cofferdam supporting structure and stress in its bracing structure exhibited good overall consistency with field monitoring data. Both sets of values were significantly below the exceedance limits specified by codes. However, certain deviations between the two persisted. Most deviations are relatively minor, with overall deviation magnitudes generally controlled within 20%. Notably, individual measurement points showed a maximum deviation reaching 34.3%. This deviation mainly stems from the fact that when the structural response values are at a relatively low level, even if the absolute deviation is quite small, the relative deviation percentage is significantly magnified. However, both the predicted values from the numerical simulation and the actual values from on-site monitoring were far below the displacement limit of 45 mm and the stress limit of 345 MPa [24, 25]. This indicated that the deviation had not affected the fundamental judgment on structural safety. For instance, the theoretical predicted deformation of the PLC construction method pile cofferdam supporting structure was 2.32 mm, while the field monitoring value was 3.10 mm, resulting in an absolute deviation of 0.78 mm but a relative deviation of 33.5%. Similarly, the predicted stress in the bracing structure was −3.84 MPa, with a monitored value of −5.15 MPa, yielding an absolute deviation of 1.31 MPa and a relative deviation of 34.1%. These cases all corroborated the above-mentioned analysis of the causes of the deviation. Consequently, comprehensive analysis confirms that the numerical simulation accurately shows the mechanical behavior of the PLC construction method pile cofferdam under varying stages, demonstrating high simulation accuracy.

## 4. Parameter sensitivity analysis based on the response surface methodology

### 4.1. Selection of structural response and sensitivity parameter

Given that high-precision static structural responses more accurately reflect the actual stress state of steel cofferdams, and lateral displacement or structural stress directly impacts their safety in deep-water areas, the maximum stress ${\overset{―}{y}}_{1}$ and displacement ${\overset{―}{y}}_{2}$ of the structure were selected as the structural response values.

In practical construction, structural parameters such as the wall thickness of steel sheet piles and steel pipe piles are prone to deviations from design specifications due to limitations in production technology and construction precision. These deviations may adversely affect critical performance indicators, including structural bearing capacity, stability, and seepage resistance. The pile bodies of the PLC construction method pile cofferdam were driven through the pebble layer and strongly weathered argillaceous siltstone, and were embedded into the moderately weathered argillaceous siltstone. The deformation of the cofferdam was predominantly controlled by pile bending. Parameter sensitivity analysis based on the response surface method can better ensure the validity of the model when the number of variables is controlled within the range of 2–7. Therefore, after comprehensive consideration, the steel sheet pile wall thickness *t*_1_, steel pipe pile wall thickness *t*_2_, horizontal spacing of internal support *l*, purlin height *h*, and bottom-sealing concrete thickness *t*_3_ were selected as the parameters for sensitivity analysis to explore their potential impacts on the structural performance of the cofferdam.

### 4.2. Response surface model construction and sensitivity analysis

Based on the selected structural responses and uncertainty parameters above, this paper employed Design Expert software to design a CCD test group and fitted response surface models for each response. The sensitivity index and sensitivity percentage for each parameter were calculated using Equations (3) and (4) to quantitatively assess the influence magnitude of each parameter on the structural response. The specific steps were as follows:

(1) Based on relevant literature [15] and practical construction experience, we established the reference values and deviation ranges for the design parameters of the PLC construction method pile cofferdam. The detailed data are provided in Table 2.

**Table 2:** Reference values and deviation ranges for the design parameters of the PLC construction method pile cofferdam.

Symbol	Design parameter	Fiducial value	Deviation range
*t*_1_/ (mm)	The steel sheet pile wall thickness	18	±20%
*t*_2_/ (mm)	The steel pipe pile wall thickness	14	±20%
*l*/ (mm)	The horizontal spacing of internal support	5015	−20%
*h*/ (mm)	The purlin height	13050	±20%
*t*_3_/ (mm)	The bottom-sealing concrete thickness	1600	±25%

(2) Based on the Central Composite Design (CCD) method, thirty-one experimental sets of five-factor two-level designs were constructed using Design-Expert software. The sample values of each parameter were then input into a finite element model for calculation, and corresponding structural response values were obtained. The related test groups and their corresponding structural response values were presented in Table 3.

**Table 3:** Related test groups and their corresponding structural response values.

The experiment group	*t*_1_ (mm)	*t*_2_ (mm)	*l* (mm)	*h* (mm)	*t*_3_ (mm)	$\mathrm{\backslash bold}{\overset{―}{y}}_{1}$ (MPa)	$\mathrm{\backslash bold}{\overset{―}{y}}_{2}$ (mm)	The experiment group	*t*_1_ (mm)	*t*_2_ (mm)	*l* (mm)	*h* (mm)	*t*_3_ (mm)	$\mathrm{\backslash bold}{\overset{―}{y}}_{1}$ (MPa)	$\mathrm{\backslash bold}{\overset{―}{y}}_{2}$ (mm)
1	14.4	11.2	5015	15660	2000	77.78	25.14	17	18	8.4	4513.5	13050	1600	108.02	32.2
2	18	14	4513.5	13050	1600	63.34	22.54	18	14.4	11.2	4012	15660	1200	101.5	30.32
3	18	14	4513.5	13050	1600	63.34	22.54	19	18	14	4513.5	13050	2400	49.03	18.66
4	14.4	16.8	5015	10440	2000	60.45	19.44	20	21.6	11.2	4012	15660	2000	69.06	23.28
5	21.6	11.2	5015	10440	2000	69.13	23.3	21	21.6	11.2	5015	15660	1200	88.92	28.23
6	21.6	16.8	5015	10440	1200	57.61	21.17	22	18	14	4513.5	13050	1600	63.34	22.54
7	10.8	14	4513.5	13050	1600	133.27	25.93	23	21.6	11.2	4012	10440	1200	89.2	28.19
8	18	14	3510.5	13050	1600	63.3	22.53	24	21.6	16.8	4012	15660	1200	57.57	21.17
9	14.4	11.2	5015	10440	1200	100.68	30.44	25	21.6	16.8	5015	15660	2000	47.89	17.22
10	18	19.6	4513.5	13050	1600	50.43	18.14	26	14.4	16.8	4012	15660	2000	60.8	19.43
11	14.4	11.2	4012	10440	2000	77.58	25.11	27	18	14	4513.5	7830	1600	63.42	22.45
12	14.4	16.8	5015	15660	1200	79.8	23.95	28	18	14	4513.5	13050	1600	63.34	22.54
13	18	14	4513.5	18270	1600	63.4	22.5	29	21.6	16.8	4012	10440	2000	47.84	17.17
14	25.2	14	4513.5	13050	1600	59.89	21.01	30	18	14	5516.5	13050	1600	63.43	22.59
15	14.4	16.8	4012	10440	1200	79.91	23.9	31	18	14	4513.5	13050	1600	63.34	22.54
16	18	14	4513.5	13050	800	76.76	28.09								

(3) The obtained structural responses were imported into Design Expert software for regression fitting of each response surface function ${\overset{―}{y}}_{i}$, with detailed results presented in Table 4.

**Table 4:** Response surface functions fitted using response surface methodology.

Undetermined coefficient	*x*_i_	$\mathrm{\backslash bold}{\overset{―}{y}}_{1}$	$\mathrm{\backslash bold}{\overset{―}{y}}_{2}$	Undetermined coefficient	*x*_i_	$\mathrm{\backslash bold}{\overset{―}{y}}_{1}$	$\mathrm{\backslash bold}{\overset{―}{y}}_{2}$
*a*	/	493.39E-00	82.42E-00	*c*_6_	*t*_2_*h	−4.2E-06	1.2E-06
*b*_1_	*t*_1_	−23.97E-00	−8.89E-01	*c*_7_	*t*_2_*t_3_	1.61E-00	1.92E-01
*b*_2_	*t*_2_	−16.22E-00	−3.58E-00	*c*_8_	l*h	5.82E-07	1.81E-08
*b*_3_	*l*	8.88E-03	5.77E-05	*c*_9_	l*t_3_	3.33E-04	−3.70E-05
*b*_4_	*h*	−5.10E-04	−3.30E-05	*c*_10_	h*t_3_	1.26E-05	5.99E-06
*b*_5_	*t*_3_	−52.92E-00	−13.79E-00	*c*_11_	*t*_1_^2	6.02E-01	17.39E-03
*c*_1_	*t*_1_*t_2_	−1.78E-01	−1.215E-02	*c*_12_	*t*_2_^2	4.42E-01	8.30E-02
*c*_2_	*t*_1_*l	3.08E-05	−4.20E-06	*c*_13_	*l*^2	−2.00E-06	−8.30E-09
*c*_3_	*t*_1_*h	−1.00E-05	9.31E-07	*c*_14_	*h*^2	−7.20E-08	−3.40E-09
*c*_4_	*t*_1_*t3	1.12E-00	7.29E-02	*c*_15_	*t*_3_^2	−3.86E-00	1.26E-00
*c*_5_	*t*_2_*l	2.36E-05	−3.60E-06				


$$\begin{array}{c} {\overset{―}{y}}_{i}=a+b_{1}t_{1}+b_{2}t_{2}+b_{3}l+b_{4}h+b_{5}t_{3}+c_{1}t_{1}t_{2}+c_{2}t_{1}l+c_{3}t_{1}h+c_{4}t_{1}t_{3}+c_{5}t_{2}l\\ +c_{6}t_{2}h+c_{7}t_{2}t_{3}+c_{8}lh+c_{9}lt_{3}+c_{10}ht_{3}+c_{11}t_{1}^{2}+c_{12}t_{2}^{2}+c_{13}l^{2}+c_{14}h^{2}+c_{15}t_{3}^{2} \end{array}$$

In the function model fitted using the response surface methodology, the interaction term coefficients directly indicated the direction and relative intensity of the interaction between parameters. A larger absolute value indicated a more significant interaction effect between the corresponding parameters. As shown in Table 3, among the interaction term coefficients *c*_1_ to *c*_10_, |*c*_7_| had the largest magnitude, followed by |*c*_4_|. This indicated that the interaction between the steel sheet pile wall thickness *t*_1_ and thickness of bottom-sealing concrete *t*_3_, as well as that between the steel pipe pile wall thickness *t*_2_ and bottom-sealing concrete thickness *t*_3_, was the most prominent.

In engineering practice, the interaction effects between parameters possess clear physical significance and engineering logic. For instance, the bottom-sealing concrete *t*_3_ provides bottom fixation and lateral restraint for the pile. When the bottom-sealing concrete *t*_3_ is insufficient, the restraint at the pile bottom weakens, making the pile shaft more prone to bending deformation. In this scenario, merely increasing the pile wall thickness (*t*_1_ or *t*_2_) has a diminished effect on improving the overall structural stiffness and stress distribution. Conversely, when the bottom-sealing concrete *t*_3_ effectively restrains the pile tip, it works synergistically with the thickened pile wall to jointly resist lateral earth and water pressure. This synergy more significantly reduces the maximum stress and displacement of the structure.

(4) Based on the dual criteria of $R^{2}$ and $R_{adj}^{2}$, we evaluated the accuracy of the fitted response surface functions was evaluated. Table 5 indicated that both $R^{2}$ and $R_{adj}^{2}$ values for each function exceeded 0.9, with a minimal difference between them. This demonstrated that the fitted functions exhibit high accuracy and could precisely characterize the relationship between design parameters and structural responses.

**Table 5:** Accuracy evaluation index of the fitting function.

Response function $\mathrm{\backslash bold}{\overset{―}{y}}_{i}$	*R* ^2^	*R*_*adj*_^2^
${\overset{―}{y}}_{1}$	0.9290	0.9148
${\overset{―}{y}}_{2}$	0.9983	0.9979

**Fig 12:**
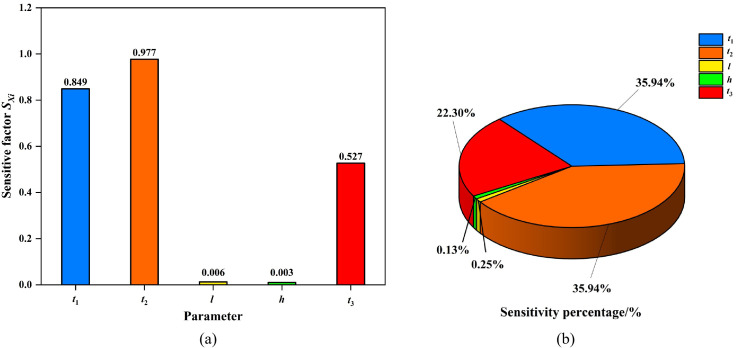
Stress sensitivity analysis of the PLC construction method pile cofferdam structure. (a) Sensitive factor $S_{x_{k}}$. (b) Sensitive percentage $\gamma_{x_{k}}$.

**Fig 13:**
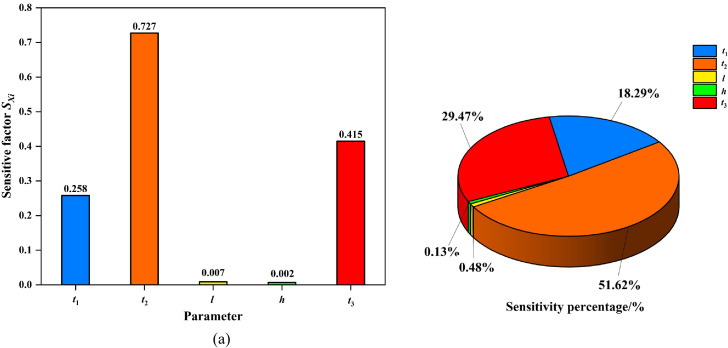
Displacement sensitivity analysis of the PLC construction method pile cofferdam structure. (a) Sensitive factor $S_{x_{k}}$. (b) Sensitive percentage $\gamma_{x_{k}}$.

(5) The sensitivity factors $S_{x_{k}}$ and sensitivity percentages $\gamma_{x_{k}}$ for each design parameter with respect to structural responses were calculated using Equations (3) and (4), based on the baseline values of each parameter. Results are presented in Table 6 and Figs 12–13.

**Table 6:** Sensitivity factor, sensitivity percentage and degree of influence of design parameters on structural response.

Design parameter	Structural response $\mathrm{\backslash bold}{\overset{―}{y}}_{1}$			Structural response $\mathrm{\backslash bold}{\overset{―}{y}}_{2}$		
	$S_{x_{k}}$	$\gamma_{x_{k}}$	The influence degree	$S_{x_{k}}$	$\gamma_{x_{k}}$	The influence degree
*t*_1_	−8.49E-01	35.94%	Major parameter	−2.58E-01	18.29%	Minor parameter
*t*_2_	−9.77E-01	41.37%	Major parameter	−7.27E-01	51.62%	Important parameter
*l*	5.98E-03	0.25%	Negligible parameter	6.76E-03	0.48%	Negligible parameter
*h*	3.12E-03	0.13%	Negligible parameter	1.79E-03	0.13%	Negligible parameter
*t*_3_	−5.27E-01	22.30%	Minor parameter	−4.15E-01	29.47%	Major parameter

As shown in Table 6, the sensitivity values of the steel pipe pile wall thickness *t*_2_ to maximum displacement and maximum stress of the cofferdam structure were 41.37% and 51.62%, respectively, classifying it as an important or major influencing parameter. For the steel sheet pile wall thickness *t*_1_ and bottom-sealing concrete thickness *t*_3_, the sensitivity values ranged from 35.94% to 18.29% and 22.30% to 29.47%, respectively, classifying them as either major parameter or minor parameters. horizontal spacing of internal support *l* and purlin height *h* exhibited negligible sensitivity (0.13%−0.48%), categorizing them as negligible parameters.

### 4.3. Comparison of sensitivity analysis results for different parameters

The single parameter method, by neglecting interaction effects among parameters, may compromise accuracy when analyzing complex structures with multi-parameter coupling. Although the response surface methodology can address this limitation, constructing accurate models necessitates a substantial number of sample points as problem complexity increases, particularly in high-dimensional spaces. Consequently, computational costs and time requirements increase significantly.

**Fig 14:**
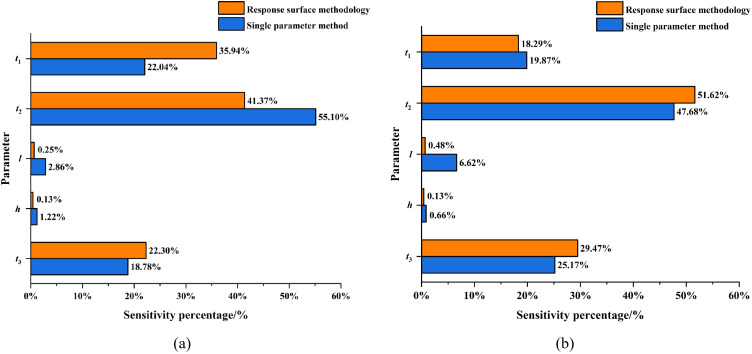
Comparison of single parameter method and response surface methodology for sensitivity analysis in the PLC construction method pile cofferdam structures. (a) Stress. (b) Displacement.

Comparing the sensitivity coefficient formula (Equation 2) for the single-parameter method with the sensitivity factor formula (Equation 3) for the response surface methodology, it is evident that both approaches quantify parameter sensitivity by evaluating the relative change between the output variable *y* and the parameter *x*. To enable direct comparison between the two analysis outcomes, the sensitivity coefficient *I* from the single-parameter method was converted into a sensitivity percentage γ’. A comparison of sensitivity percentages for parameters computed by the two methods is presented in Table 7 and Fig 14.

**Table 7:** Sensitivity of the design parameters to the stress and displacement of the structure.

Project		*t*_1_	*t*_2_	*l*	*h*	*t*_3_
Stress sensitivity percentage	$\gamma_{x_{i}}^{\prime }$	22.04%	55.10%	2.86%	1.22%	18.78%
	$\gamma_{x_{i}}$	35.94%	41.37%	0.25%	0.13%	22.30%
Displacement sensitive percentage	$\gamma_{x_{i}}^{\prime }$	19.87%	47.68%	6.62%	0.66%	25.17%
	$\gamma_{x_{i}}$	18.29%	51.62%	0.48%	0.13%	29.47%

As can be seen from Table 7 and Fig 14, when the single parameter method and the response surface methodology were used to analyze the sensitivity of the stress and displacement parameters of the PLC pile cofferdam structure, the ranking of each parameter’s sensitivity was consistent. However, there were some differences in the quantification of sensitivity. For instance, in the stress sensitivity analysis of cofferdam structure, the single parameter method yielded a sensitivity percentage for the steel sheet pile wall thickness t₁ that was 13.9% lower than that obtained via response surface methodology. This discrepancy caused *t*₁ to be classified as a minor parameter in the single parameter method but a major parameter in response surface methodology. Conversely, the sensitivity percentage of the steel pipe pile wall thickness *t*_2_ was 13.73% higher in the single-parameter method compared with the response surface methodology. This discrepancy caused *t*_2_ to be identified as an important parameter in the single parameter analysis, whereas it was classified as a major parameter in the response surface methodology.

As analyzed in Section 3.2, the steel sheet pile wall thickness *t*_1_, steel pipe pile wall thickness *t*_2_ and bottom-sealing concrete thickness *t*_3_ significantly affected the maximum displacement and maximum stress of the cofferdam structure, necessitating strict control during construction. Conversely, the horizontal spacing of internal support *l* and purlin height *h* had minimal effects on these structural responses and could be considered negligible parameters, requiring less attention during construction.

## 5. Multi-objective optimization

Different parameter combinations induce complex nonlinear characteristics in the stress distribution and displacement change of the cofferdam. Previous analyses revealed that the maximum stress and displacement of the structure, calculated based on the design reference values for the steel sheet pile wall thickness *t*_1_, steel pipe pile wall thickness *t*_2_, and bottom-sealing concrete thickness *t*_3_, are significantly lower than the limits specified in relevant codes (the maximum stress value for the cofferdam steel structure is 345 MPa, the displacement limit is L/400 = 45 mm, and the concrete tensile stress limit is 1.43 MPa) [24–26]. This resulted in poor economic performance. Accordingly, in this subsection, the NSGA-II algorithm was employed to conduct a multi-objective optimization study focusing on three key parameters: the steel sheet pile wall thickness *t*_1_, steel sheet pile wall thickness *t*_2_, and bottom-sealing concrete thickness *t*_3_. The objectives are to ensure rational structural stress distribution and effective displacement control while pursuing optimal economic performance.

### 5.1. Multi-objective optimization model

According to relevant specifications and construction experience [27], the steel sheet pile wall thickness *t*_1_ and steel pipe pile wall thickness *t*_2_ in deep-water areas shall be no less than 12 mm and 10 mm, respectively. Theoretical formula calculations indicated that, while satisfying anti-floating stability and structural strength design requirements, the minimum bottom-sealing concrete thickness *t*_3_ for the PLC construction method pile cofferdam at the Luoxizhou Super Bridge main pier should not be less than 1.37 m. In actual design, the design values for the steel sheet pile wall thickness *t*_1_, the steel pipe pile wall thickness t2, the bottom-sealing concrete thickness t3 were 18 mm, 14 mm, and 1600 mm respectively. After comprehensive consideration, we took each parameter’s design value as the central point and used the absolute difference between the code-specified minimum value and the design value as the radius, the optimization intervals for each parameter were determined as follows: the steel sheet pile wall thickness *t*_1_ [12 mm, 24 mm], steel pipe pile wall thickness *t*_2_ [10 mm, 18 mm], and bottom-sealing concrete thickness *t*_3_ [1370 mm, 1830 mm].

During the analysis of previous experimental group data, it was observed that the maximum stress in the PLC construction method pile cofferdam generally remained below 200 MPa, indicating a significant safety margin compared to the 345 MPa yield strength, and that a positive correlation existed between maximum stress and displacement. Additionally, due to the inadequate tensile strength of concrete, we used the economic cost *f*_1_, the maximum displacement of the PLC construction method pile cofferdam *f*_2_, and the maximum tensile stresses in the bottom-sealing concrete *f*_3_ as objective functions for multi-objective optimization. Additionally, the core parameters and assumptions of the economic cost assessment model for the PLC construction method pile cofferdam were as follows: The initial purchase costs per ton for steel sheet piles (101 t), steel pipe piles (231 t), and internal supports (61 t) were set at 8,500 yuan/t, 6,200 yuan/t, and 6,200 yuan/t, respectively. The unit cost of bottom-sealed concrete (932 m^3^) was 600 yuan/m^3^. The residual value of steel material recycling was set at 2,000 yuan/t. However, installation and dismantling costs were not taken into account.

Based on response surface methodology, explicit expressions for the objective functions *f*_1_, *f*_2_ and *f*_3_ were regressed and fitted using sensitivity parameters *t*_1_, *t*_2_, and *t*_3_, thereby a multi-objective mathematical optimization model was constructed as presented in Equation (6). This study employed the NSGA-II algorithm to solve the multi-objective optimization problem. The algorithm utilized a constraint domination principle to evaluate the feasibility of individuals without incorporating penalty functions. This model is formulated to minimize the economic cost *f*_1_ while ensuring rational stress distribution and effective displacement control within the structure.


$$\begin{array}{c} {}*20c\min=[f_{1}(x_{1},x_{2},x_{3}),f_{2}(x_{1},x_{2},x_{3}),f_{3}(x_{1},x_{2},x_{3})]\\ 12mm\leq x_{1}\leq 24mm\\ 10mm\leq x_{2}\leq 18mm\\ 1370mm\leq x_{3}\leq 1830mm\\ f_{2}\leq 45mm\\ f_{3}\leq 1.43MPa \end{array}\}$$ (6)

### 5.2. Pareto solution

The NSGA-II algorithm had a population size of 50, a crossover probability of 0.8 and a mutation probability of 0.2. The number of iterations was 100. After 46 iterations, the Pareto front stabilized, indicating good convergence and stability of the solution set, and that the Pareto front obtained by multiple runs was essentially identical. Fig 8 illustrates the Pareto optimal solution set, the initial population, the CCD experimental design groups, and the spatial distribution of their corresponding solutions.

**Fig 15:**
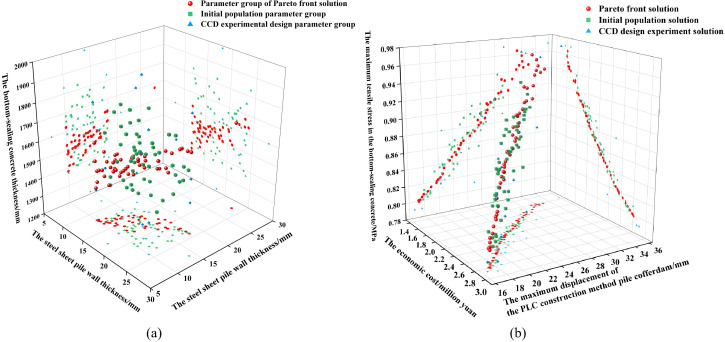
The spatial distribution of Pareto optimal solution set, initial population, CCD experimental design group and their corresponding solutions. (a) The distribution of parameter groups. (b) The distribution of solutions.

Fig 15(a) shows that the initial population is relatively dispersed in space, exhibiting good diversity. This allows it to cover a larger range of the solution space, facilitating better exploration of different regions during the global search process and reducing the risk of converging to local optima. Simultaneously, the optimized Pareto optimal solution set is relatively concentrated. This indicates that the NSGA-II algorithm demonstrates good convergence and distribution characteristics during the optimization process, forming a relatively clear outline. Fig 15(b) further corroborates this conclusion. It shows that the Pareto optimal solutions are more concentrated compared to the initial population solutions, forming a distinct curve. This demonstrates that the algorithm effectively concentrates the solutions along the non-dominated front.

The Pareto frontier solutions illustrated the trade-off relationships among the three optimization objectives: the maximum displacement of the PLC construction method pile cofferdam *f*_2_, maximum tensile stress in the bottom-sealing concrete *f*_3_, and economic cost *f*_1_. As the values of the first two optimization objectives (*f*_2_ and *f*_3_) decreased, the economic cost *f*_1_ increased accordingly. This reflected the contradictory relationship between structural performance enhancement and economic cost increase, suggesting that decision-makers should comprehensively weigh the balance among the three objectives to select the optimal solution that best meets the project’s requirements. Furthermore, the projection of the Pareto frontier solutions onto the XY plane demonstrated a positive correlation between the maximum tensile stress in the bottom-sealing concrete *f*_3_ and maximum displacement of the PLC construction method pile cofferdam *f*_2_. This observation highlighted the critical importance of simultaneously monitoring the stress state of the bottom-sealing concrete during displacement control, thereby ensuring the overall safety and stability of the structure.

The maximum tensile stress in the bottom-sealing concrete *f*_3_ and the maximum displacement of the PLC construction method pile cofferdam *f*_2_ both exhibit a nonlinear decreasing trend with increasing the economic cost *f*_1_. This indicates that the marginal utility of increasing the economic cost for improving structural performance diminishes. This finding implies that in the low-cost scenario, a relatively small investment can lead to a significant improvement in structural performance. Conversely, in the high-cost range, even a substantial financial input results in only very limited further enhancement of structural performance.

### 5.3. Decision result

In the field of multi-objective optimization, each point on the Pareto frontier represents an optimal solution to an optimization model, corresponding to a unique set of optimization schemes. In view of this, this study introduced a multi-criteria decision-making method: the entropy-weighted TOPSIS method. The core of this method lay in quantifying the superiority or inferiority of these schemes by calculating the Euclidean distances from each optimization scheme to both the ideal solution and the negative-ideal solution [28–30]. To comprehensively evaluate the performance of the optimized schemes and provide a more substantive basis for decision-making, this study incorporated initial design scheme into the evaluation framework. Specifically, the entropy-weighted TOPSIS method was employed to assess both the initial design scheme and the Pareto frontier solutions. The detailed implementation steps were outlined.

**Fig 16:**
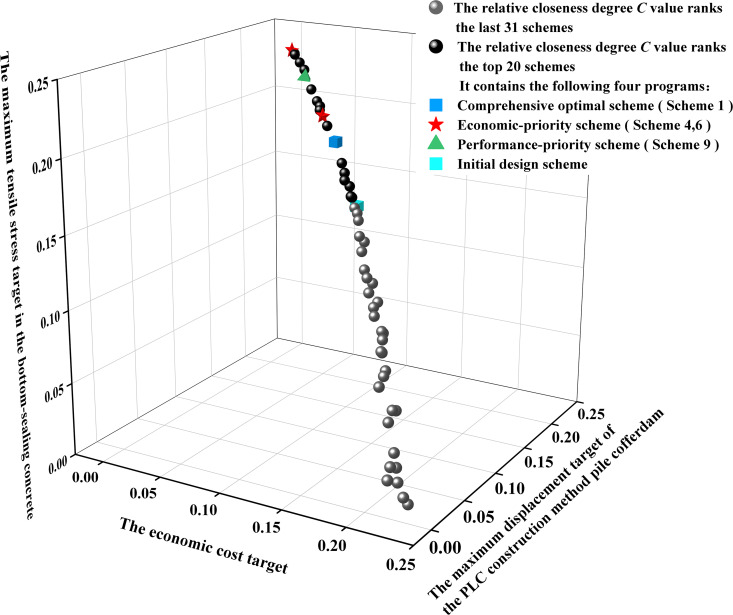
The spatial distribution of the optimization scheme and the initial design scheme after sum-of-squares normalization.


(1) Data positive orientation and standardization processing: To ensure comparability among objectives and improve the rationality and versatility of decision-making, it was necessary to ensure consistency in the evaluation directions of each objective function. Given that all objective functions $f_{1}$, $f_{2}$, and $f_{3}$ are minimization objectives, they were transformed into a maximization direction using Equation (7) to standardize the optimization orientation. Subsequently, sum-of-squares normalization was applied using Equation (8) to eliminate dimensional differences, resulting in the construction of a normalized matrix z. The normalized Pareto frontier solutions and initial design solution distribution are illustrated in Fig 16.

$$f_{ij}^{\prime }=\frac{\max(f_{ij})-f_{ij}}{\max(f_{ij})-\min(f_{ij})}$$ (7)

$$z_{ij}=\frac{f_{ij}^{\prime }}{\sqrt{\sum_{i=1}^{n}f{{}_{ij}^{\prime }}^{2}}}$$ (8)

In the equation, $f_{ij}$, $f_{ij}^{\prime }$ and $z_{ij}$ represent the objective function value of the *i*-th scheme for the *j*-th objective, the target value after positive orientation processing, and the sum-of-squares normalized target value, respectively. $\max(f_{ij})$ and $\min(f_{ij})$ denote the maximum and minimum values of the *j*-th objective function.

(2) Weight value calculation: To address the subjective bias in weight allocation during multi-objective decision-making, the entropy-weight method was introduced to objectively quantify the weights ω of objectives.

① The proportion $P_{ij}$ of the function value was calculated for the *i*-th optimization scheme under the *j*-th objective.


$$P_{ij}=f_{ij}/(\sum_{i=1}^{n}f_{ij})$$ (9)

② The information entropy E was calculated for each objective.


$$e_{j}=-(\sum_{i=1}^{n}\left(P_{ij}\cdot \ln(P_{ij})\right))/\ln(n)$$ (10)

③ The weight coefficient $\omega_{j}$ was calculated for each objective.


$$\omega_{j}=(1-e_{j})/(\sum_{i=1}^{n}\left(1-e_{j}\right))$$ (11)

The weight ω for each objective is presented in Table 8. It is evident that the weights among the objectives are basically consistent. This indicates that the importance of these three objectives for distinguishing the advantages and disadvantages of the alternatives was approximately equal. Consequently, the ultimately selected “optimal solution” is inevitably one that achieves a high degree of balance among these three factors, rather than being heavily skewed towards any single one. This suggests decision-makers to avoid subjective preferences and seek a truly balanced optimal design.

**Table 8:** Weights of each objective.

Target	Information entropy value *e*	Information utility value *d*	Weight coefficient *w*
The economic cost *f*_1_	0.9560	0.0440	33.34%
The maximum displacement of the PLC construction method pile cofferdam *f*_2_	0.9543	0.0457	34.57%
The maximum tensile stress in the bottom-sealing concrete *f*_3_	0.9576	0.0424	32.09%

(3) Calculation and Ranking of Relative Closeness Degree C: Using the weight values ω, the distances of each optimization scheme from the positive ideal solution $D^{+}$ and the negative ideal solution $D^{-}$ were calculated. Subsequently, the relative closeness degree C was determined. All optimization schemes were ranked based on their C values. A higher $C_{i}$ value indicates that the optimization scheme is closer to the optimal solution.


$$D_{i}^{+}=\sqrt{\sum_{j=1}^{m}\omega_{j}{\left(z_{ij}-z_{j}^{+}\right)}^{2}}$$ (12)

$$D_{i}^{-}=\sqrt{\sum_{j=1}^{m}\omega_{j}{\left(z_{ij}-z_{j}^{-}\right)}^{2}}$$ (13)

$$C_{i}=\frac{D_{i}^{-}}{D_{i}^{+}+D_{i}^{-}}$$ (14)

In the above equation, $z_{j}^{+}$ and $z_{j}^{-}$ represent the ideal solution and the negative-ideal solution for the *j*-th objective, respectively.

The top 20 relatively optimal solutions (including design scheme), ranked by the relative closeness degree, are presented in Table 9.

**Table 9:** The relative optimal scheme of the top 20 relative closeness degree.

No.	*t*_1_ (mm)	*t*_2_ (mm)	*t*_3_ (mm)	*f*_1_ (million yuan)	*f*_2_ (mm)	*f*_3_ (MPa)	Relative Closeness degree *C*
1	16.59	14.62	1745.57	2.287	21.60	0.86	0.615
2	15.39	15.22	1656.00	2.270	21.97	0.86	0.614
3	18.30	15.22	1440.48	2.363	22.07	0.84	0.614
4	13.48	11.60	1398.63	1.839	29.23	0.91	0.613
5	18.99	14.80	1380.64	2.361	22.64	0.84	0.613
6	13.32	16.42	1549.35	2.293	22.43	0.85	0.612
7	22.55	11.30	1621.37	2.104	25.53	0.88	0.61
8	12.37	14.76	1779.37	2.162	23.07	0.88	0.608
9	23.61	17.91	1539.48	2.774	17.87	0.80	0.608
10	20.99	15.34	1373.98	2.480	21.47	0.82	0.607
11	21.71	17.08	1703.25	2.672	18.04	0.81	0.604
12	16.90	11.32	1475.29	1.919	27.83	0.90	0.604
13	17.73	11.34	1722.86	1.984	25.95	0.91	0.601
14	21.39	10.10	1683.36	1.957	27.57	0.91	0.601
15	21.56	14.09	1647.62	2.363	21.34	0.84	0.599
16	13.17	11.07	1679.70	1.777	28.41	0.94	0.599
17	21.27	17.59	1487.40	2.677	18.85	0.81	0.596
18	17.46	12.57	1415.77	2.077	25.96	0.87	0.591
19/Initial design scheme	18.00	14.00	1600.00	2.228	22.66	0.86	0.591
20	17.21	13.21	1551.59	2.120	24.30	0.87	0.59

Under the multi-objective optimization framework for the PLC construction method pile cofferdam, each solution on the Pareto front corresponds to an optimal construction scheme. Based on the performance indicator of the economic cost *f*_1_, the maximum displacement of the PLC construction method pile cofferdam *f*_2_, and the maximum tensile stress in the bottom-sealing concrete *f*_3_ for each optimized construction scheme, a normalized performance relative closeness index was constructed to quantitatively assess the comprehensive performance of each scheme within the multi-objective optimization space. For example, when comparing Alternative 1 and Alternative 3, we no longer simply make a statement about their relative advantages and disadvantages. Instead, we conduct an analysis from the perspective of multi-criteria decision-making. Although Alternative 1 has a 2.32% higher maximum tensile stress in the bottom-sealing concrete compared to Alternative 3, it simultaneously achieves a 3.25% reduction in the economic cost and a 2.13% decrease in the maximum displacement of the PLC construction method pile cofferdam. This indicates that, within the framework of multi-objective optimization, Alternative 1 represents a more balanced and efficient trade-off. The minor sacrifice in concrete tensile stress is compensated by the gains in cost control and deformation safety, which is engineeringly feasible.

As shown in Table 9, Scheme 1 (C = 0.615) emerged as the comprehensive optimal solution, positioned at the highest level of the Pareto frontier non-dominated solution set due to its highest relative closeness degree. Compared with the initial design scheme, this scheme achieved a balanced optimization of structural performance and economy: the economic cost *f*_1_ increased by only 2.63%, the maximum displacement of the PLC construction method pile cofferdam *f*_2_ was reduced by 4.68%, and the maximum tensile stress in the bottom-sealing concrete *f*_3_ remained essentially unchanged. Scheme 9 (C = 0.610) is a performance-priority scheme. Compared with the design scheme, its economic cost *f*_1_ increased by 24.5% to ¥2.7737 million, while the maximum displacement of the PLC construction method pile cofferdam *f*_2_ decreased to 17.87 mm (a reduction of 21.25%) and maximum tensile stress in the bottom-sealing concrete *f*_3_ dropped to 0.80MPa (a reduction of 6.88%). This indicated that under the condition of a relatively significant increase in economic cost *f*_1_, the structural performance had been significantly improved. Scheme 4 (C = 0.613) and Scheme 16 (C = 0.599) represented economy-priority schemes. Both achieved significant reductions in economic cost *f*_1_ (decreases of 20.22% and 17.44% respectively) through parameter combinations approaching the lower limits of design specifications. However, this resulted in increased structural deformation and stress: the maximum displacement of the PLC construction method pile cofferdam *f*_2_ rise to 29.23 mm and 28.41 mm (increases of 29.00% and 25.38%), while the maximum tensile stresses in the bottom-sealing concrete *f*_3_ increased to 0.91MPa and 0.94MPa (increases of 5.78% and 9.08%).

Table 9 indicated that the maximum displacement of the PLC construction method pile cofferdam *f*_2_ and the maximum tensile stress of the bottom-concrete *f*_3_ ranged from 17.87 mm to 29.23 mm and 0.80MPa to 0.94MPa, respectively, and both meet the code requirements (displacement limit L/400 = 45 mm, tensile stress limit 1.43MPa) [24,26]. This demonstrates that both Scheme 4 and Scheme 16 possess significant safety margins in structural performance.

The low ranking of the design scheme in the relative closeness ranking (C = 0.591, ranked 19th) revealed the limitations of traditional empirical design methodologies in addressing multi-objective trade-offs, global parameter optimization, and quantitative decision-making. This highlighted the practical value of the NSGA-II algorithm in optimizing PLC construction method pile cofferdams.

**Fig 17:**
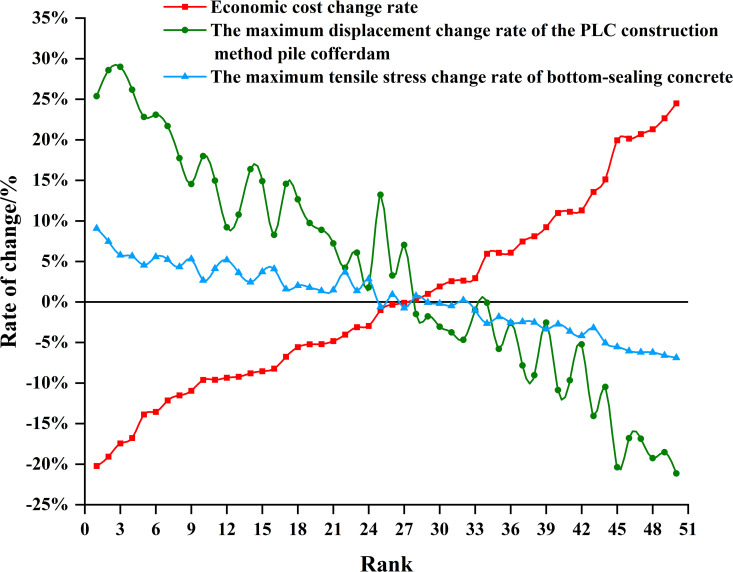
A comparison of the objective function values for the optimization schemes and design scheme.

To investigate the influence of economic cost *f*_1_ changes on the maximum displacement of the PLC construction method pile cofferdam *f*_2_ and maximum tensile stress in the bottom-sealing concrete *f*_3_, the optimization schemes were sorted by economic cost from low to high. Subsequently, the objective function values of these schemes were compared with those of the benchmark design. The results are presented in Fig 17.

As depicted in Fig 17, within the Pareto frontier solutions, both the maximum displacement of the PLC construction method pile cofferdam *f*_2_ and the maximum tensile stresses in the bottom-sealing concrete *f*_3_ exhibited a simultaneous decreasing trend with increasing economic cost *f*_1_. Furthermore, variations in economic cost *f*_1_ significantly influenced the maximum displacement of the PLC construction method pile cofferdam *f*_2_, while exerted minimal impact on the maximum tensile stresses in the bottom-sealing concrete. *f*_3_

## 6. Conclusion

Based on the PLC construction method pile cofferdam of Luoxizhou Super Bridge, this study conducted a parameter sensitivity analysis using the response surface methodology. Leveraging the identified sensitive parameters, a multi-objective optimization was performed targeting the economic cost, maximum displacement of the PLC construction method pile cofferdam, and maximum tensile stress in the bottom-sealing concrete via the NSGA-II algorithm. The conclusions and prospects are as follows:

(1) The steel pipe pile wall thickness *t*_2_ demonstrates the highest sensitivity to maximum displacements and maximum stresses in the cofferdam structure, thus serving as important parameter or major parameter. The steel sheet pile wall thickness *t*_1_ and bottom-sealing concrete thickness *t*_3_ exhibit moderate sensitivity to structural responses, categorizing them as major parameter or minor parameter. Sensitivity percentages for all other parameters remain below 5%, rendering them negligible. The parameter sensitivity ranking obtained using the response surface methodology was similar to that from the single parameter method, but the former enabled more accurate quantification for PLC construction method pile cofferdams.
(2) Compared to the initial design scheme, Scheme 4 and Scheme 16 significantly reduced the economic cost. The corresponding maximum displacement of the PLC construction method pile cofferdam were 29.23 mm and 28.41 mm, respectively, while the maximum tensile stresses in the bottom-sealing concrete were 0.91 MPa and 0.94 MPa, respectively. These values all satisfied the specification requirements (displacement limit: L/400 = 45 mm; tensile stress limit: 1.43 MPa). This indicates substantial safety margins for both structural performance. Therefore, they could be adopted as cost-effective design alternatives prioritizing economy.
(3) The entropy-weight TOPSIS decision-making analysis indicates that traditional empirical design yielded a lower closeness ranking due to deficiencies in multi-objective trade-offs, global parameter search, and quantitative decision-making. In contrast, the NSGA-II algorithm achieves a nonlinear cooperative relationship between economic cost and structural performance through multi-objective optimization. Specifically, as economic cost increase, the maximum displacement of the PLC construction method pile cofferdam decreases substantially, while the reduction rate of the maximum tensile stresses in the bottom-sealing concrete exhibits a relatively slower decline. This finding provides critical quantitative decision-making support for optimizing the design of PLC construction method pile cofferdams.
(4) Due to the limitations of the study scope and conditions, this research did not take into account the degree of influence exerted by factors such as soil stiffness, water flow velocity, and construction errors on the PLC sheet pile cofferdam. Given this, future researchers may further broaden the research scope when conducting related analyses, in order to achieve more comprehensive and in-depth research results.

## References

[1] Ren Y, Yang S, Zhou M, Zhang X, Li J, Tian Y. Case study: design optimization and field tests of a large geotextile mat cofferdam combined with steel sheet piles. Geotext Geomembranes. 2025;53(6):1257–65. doi: 10.1016/j.geotexmem.2025.05.004

[2] Li F, Huang J. Deformation law and stability state of cofferdam during pumping process in PC combined method pile cofferdam. Desalinat Water Treatment. 2023;313:315–30. doi: 10.5004/dwt.2023.30030

[3] Zhu Y, Bi J, Xing H, Peng M, Huang Y, Wang K, et al. Stability analysis of cofferdam with double-wall steel sheet piles under wave action from storm surges. Water. 2024;16(8):1181. doi: 10.3390/w16081181

[4] Liu J, Wang D. Static parameter sensitivity analysis of long-span cable-stayed bridge based on RSM. J Highway Transp Res Dev (English Ed). 2016;10(1):64–71. doi: 10.1061/jhtrcq.0000487

[5] Zhan Y, Huang Y, Fan Z, Shao J, Qin Z. Sensitivity analysis of asymmetric steel truss cable-stayed bridge based on response surface method. Railway Engineering. 2023;63(10):62–8.

[6] Hooshmandi S, Kioumarsi B, Kioumarsi M, Hajmohammadian Baghban M. Application of response surface method (RSM) on sensitivity analysis of reinforced concrete bridge pier wall. In: NORDIC CONCRETE RESEARCH. Aalborg, Denmark;Norsk Betongforening. 2017.

[7] Chen F, Li Y, Luo J, Zhang W, He Y. Sensitivity analysis of disease causes in PC box girder bridges based on response surface method. 2024;64(06):90–5.

[8] Hu H, Wu H, Sun Q. Response analysis of hybrid girder cable-stayed bridge in cantilever assembly optimization based on the response surface method. Forest Eng. 2022;38(06):115–23.

[9] Mao J, Liu X, Yan T, Duan P, Chen W. Analysis of the mechanical behaviour of double-wall steel boxed cofferdam structures. JESTR. 2024;17(1):199–205. doi: 10.25103/jestr.171.23

[10] Du G, Zhao X, Xu D, Yang P. Research on steel sheet pile cofferdam based on stress seepage coupling analysis. Adv Machinery Materials Sci Eng Application X. 2024;:367–73.

[11] Wang Q, Li C, Ma Y, Hu Z, Lv H, Liu W. Research on deformation characteristics and design optimization of super-large cofferdam enclosure structure. Buildings. 2023;13(10):2429. doi: 10.3390/buildings13102429

[12] Li P, Sun X, Chen J, Shi J. Effects of new construction technology on performance of ultralong steel sheet pile cofferdams under tidal action. Geomechan Eng. 2021;27(6):561–71.

[13] Jiang Z, Yang C, Yue H. Multi-objective optimization of steel pipe pile cofferdam construction based on improved sparrow search algorithm. Appl Sci. 2024;14(22).

[14] Shao J, Fan Z, Huang Y, Zhan Y, Cai Q. Multi-objective optimization of double-walled steel cofferdams based on response surface methodology and particle swarm optimization algorithm. Structures. 2023;49:256–66. doi: 10.1016/j.istruc.2023.01.092

[15] Wan Y. Analysis of Stress and Deformation Characteristic of PC Construction Method Pile Cofferdam and Optimization of Support Research. Lanzhou: Lanzhou Jiaotong University. 2024.

[16] Papanikolaou N, Anyfantis K. Construction of surrogate models for predicting the buckling strength of stiffened panels through DoE and RSM methods. EC. 2021;39(4):1374–406. doi: 10.1108/ec-03-2021-0176

[17] Keshtegar B, Nehdi ML, Trung N-T, Kolahchi R. Predicting load capacity of shear walls using SVR–RSM model. Applied Soft Computing. 2021;112:107739. doi: 10.1016/j.asoc.2021.107739

[18] Tian Z, Zhang W, Cai Y, Lin L, Yang Y. Segmented real-time uncertainty-based updating of finite element model of cantilever casting arch bridge. J Shenyang Univers Technol. 2025;47(03):398–408.

[19] Boukhatem G, Bencheikh M, Benzerara M, Anas SM, Sabri MM, Najm HM. Optimizing properties of clayey soil using lime and waste marble powder: a sustainable approach for engineering applications. Front Materials. 2024;11:1392875.

[20] Wang Z, Sun S, Ding Y. Fatigue optimization of structural parameters for orthotropic steel bridge decks using RSM and NSGA-II. Mathematical Probl Eng. 2022;2022(1):4179898.

[21] Xiang Z, Zhu Z. Multi-objective optimization of a composite orthotropic bridge with RSM and NSGA-II algorithm. J Constructional Steel Res. 2022;188:106938. doi: 10.1016/j.jcsr.2021.106938

[22] Li Z, Qi J, Wang J. Multi-objective optimization design of PCS box girder bridges with small and medium spans using genetic algorithms. Buildings. 2025;15(3):361. doi: 10.3390/buildings15030361

[23] CCCC HIGHWAY CONSULTANTS CO., LTD. Ministry of Housing and Urban-Rural Development of the People’s Republic of China. General Specifications for Design of Highway Bridges and Culverts: JTG D60-2015[S]. Beijing. China Communications Press. 2015.

[24] Ministry of Housing and Urban-Rural Development of the People’s Republic of China. Technical standard for monitoring of building excavation engineering: GB 50497-2019. Beijing: China Planning Publishing House. 2019.

[25] Ministry of Housing and Urban-Rural Development of the People’s Republic of China. Standard for design of steel structures: GB 50017-2017. Beijing: China Architecture & Building Press. 2017.

[26] Ministry of Housing and Urban-Rural Development of the People’s Republic of China. Code for design of concrete structures: GB/T 50010-2010. Beijing: China Architecture & Building Press. 2010.

[27] Ministry of Housing and Urban-Rural Development of the People’s Republic of China. Technical standard of steel cofferdam engineering: GB/T 51295-2018. Beijing: China Planning Publishing House. 2018.

[28] Wang M, Chen C, Fan B, Yin Z, Li W, Wang H, et al. Multi-objective optimization of envelope design of rural tourism buildings in southeastern coastal areas of china based on NSGA-II algorithm and entropy-based TOPSIS method. Sustainability. 2023;15(9):7238. doi: 10.3390/su15097238

[29] Yang H, Liu N, Li M, Gu M, Gao Q. Design and optimization of heat pipe-assisted liquid cooling structure for power battery thermal management based on NSGA-II and entropy weight-TOPSIS method. Applied Thermal Engineering. 2025;272:126416. doi: 10.1016/j.applthermaleng.2025.126416

[30] Jiang R, Ci S, Liu D, Cheng X, Pan Z. A hybrid multi-objective optimization method based on NSGA-II algorithm and entropy weighted topsis for lightweight design of dump truck carriage. Machines. 2021;9(8):156. doi: 10.3390/machines9080156

